# Risk Factors for Invasive Aspergillosis in Patients Admitted to the Intensive Care Unit With Coronavirus Disease 2019: A Multicenter Retrospective Study

**DOI:** 10.3389/fmed.2021.753659

**Published:** 2021-11-16

**Authors:** Jiqian Xu, Xiaobo Yang, Zheng Lv, Ting Zhou, Hong Liu, Xiaojing Zou, Fengsheng Cao, Lu Zhang, Boyi Liu, Wei Chen, Yuan Yu, Huaqing Shu, Shiying Yuan, Ming Hu, Chaolin Huang, You Shang

**Affiliations:** ^1^Department of Critical Care Medicine, Union Hospital, Tongji Medical College, Huazhong University of Science and Technology, Wuhan, China; ^2^Research Center for Translational Medicine, Jinyintan Hospital, Wuhan, China; ^3^Institute of Anesthesiology and Critical Care Medicine, Union Hospital, Tongji Medical College, Huazhong University of Science and Technology, Wuhan, China; ^4^Department of Critical Care Medicine, Xiangyang Central Hospital, Affiliated Hospital of Hubei University of Arts and Science, Xiangyang, China; ^5^Department of Critical Care Medicine, Taihe Hospital Affiliated to Hubei University Medicine, Shiyan, China; ^6^Department of Critical Care Medicine, Wuhan Pulmonary Hospital, Wuhan, China

**Keywords:** COVID-19, CAPA, methylprednisolone, mortality, thrombocytopenia

## Abstract

**Background:** Invasive pulmonary aspergillosis (IPA) is a life-threatening complication in coronavirus disease 2019 (COVID-19) patients admitted to intensive care units (ICUs), but risk factors for COVID-19-associated IPA (CAPA) have not been fully characterized. The aim of the current study was to identify factors associated with CAPA, and assess long-term mortality.

**Methods:** A retrospective cohort study of adult COVID-19 patients admitted to ICUs from six hospitals was conducted in Hubei, China. CAPA was diagnosed via composite clinical criteria. Demographic information, clinical variables, and 180-day outcomes after the diagnosis of CAPA were analyzed.

**Results:** Of 335 critically ill patients with COVID-19, 78 (23.3%) developed CAPA within a median of 20.5 days (range 13.0–42.0 days) after symptom onset. Compared to those without CAPA, CAPA patients were more likely to have thrombocytopenia (50 vs. 19.5%, *p* < 0.001) and secondary bacterial infection prior to being diagnosed with CAPA (15.4 vs. 6.2%, *p* = 0.013), and to receive vasopressors (37.2 vs. 8.6%, *p* < 0.001), higher steroid dosages (53.9 vs. 34.2%, *p* = 0.002), renal replacement therapy (37.2 vs. 13.6%, *p* < 0.001), and invasive mechanical ventilation (57.7 vs. 35.8%, *p* < 0.001). In multivariate analysis incorporating hazard ratios (HRs) and confidence intervals (CIs), thrombocytopenia (HR 1.98, 95% CI 1.16–3.37, *p* = 0.012), vasopressor use (HR 3.57, 95% CI 1.80–7.06, *p* < 0.001), and methylprednisolone use at a daily dose ≥ 40 mg (HR 1.69, 95% CI 1.02–2.79, *p* = 1.02–2.79) before CAPA diagnosis were independently associated with CAPA. Patients with CAPA had longer median ICU stays (17 days vs. 12 days, *p* = 0.007), and higher 180-day mortality (65.4 vs. 33.5%, *p* < 0.001) than those without CAPA.

**Conclusions:** Thrombocytopenia, vasopressor use, and corticosteroid treatment were significantly associated with increased risk of incident IPA in COVID-19 patients admitted to ICUs. The occurrence of CAPA may increase the likelihood of long-term COVID-19 mortality.

## Introduction

Coronavirus disease 2019 (COVID-19) is caused by severe acute respiratory syndrome coronavirus 2 (SARS-CoV-2) infection, and has now been responsible for millions of hospitalizations (1, 2). Most patients recover from COVID-19 but some—mainly critically ill patients—are vulnerable to dangerous secondary infection by opportunistic pathogens (3–5). Among the relevant microbes, *Aspergillus* spp. is considered very serious and is potentially deadly, particularly if it induces invasive pulmonary aspergillosis (IPA) (3, 6, 7).

COVID-19-associated IPA (CAPA) is a life-threatening complication in intensive care unit (ICU) patients, and the reported incidence of CAPA varies from 4 to 35% (4, 5, 8–13). The variation in estimates may be partly due to different diagnostic approaches and definitions (3, 4, 6). Regardless of the definition used, IPA is reportedly associated with both viral and host factors (3–6). For example, SARS-CoV-2 can induce severe lung or immune damage that impairs the host's ability to clear the *Aspergillus* spp. Some critical patients have been immunocompromised due to diabetes and chronic kidney disease (14), which are associated with an increased risk of developing IPA (4, 14). Some drugs used to treat COVID-19 including steroids may also render patients more vulnerable to *Aspergillus* spp. infection (15). Notably however, few studies have focused on these host and infection variables in an effort to facilitate early stratification of COVID-19 patients at high risk of developing CAPA.

The current study was conducted to determine the incidence of CAPA in COVID-19 patients admitted to ICUs, and investigate risk factors associated with CAPA occurrence and long-term outcomes.

## Methods

### Study Design and Participants

This retrospective study was conducted at six ICUs of Union Hospital, Union Hospital West Campus, Jinyintan Hospital, Wuhan Pulmonary Hospital, Xiangyang Central Hospital, and Taihe Hospital in China. Patients aged ≥ 18 years were screened if they had a confirmed diagnosis of SARS-CoV-2 infection between 29 December 2019 and 01 April 2020. The ICU admission criteria and treatment decisions for all patients were as previously described (16, 17). Patients were tested for invasive fungal infections if they were suspected of having IPA or there was evidence of clinical disease progression while they were in the ICU (12). All patients with one or more mycological tests of serum or bronchoalveolar lavage (BAL), bronchial aspiration (BA), galactomannan culture, or *Aspergillus* spp. PCR were analyzed as potential cases.

CAPA was diagnosed if *Aspergillus* spp. was cultured from the patient's BAL, or if their serum galactomannan index was ≥0.5, or if two of the following three conditions were met in accordance with recent CAPA studies (6, 11, 12): Positive *Aspergillus* spp. culture of BA, BAL galactomannan index ≥ 1.0, and positive BAL *Aspergillus fumigatus* qPCR. In patients without CAPA during hospitalization, the day of IPA diagnosis was identified as the day on which the first diagnostic microbiological test after ICU admission was performed. In all six hospitals galactomannan index testing was performed using the Bio-Rad enzyme linked immunosorbent assay (ELISA) test. Cases in which the only positive mycological evidence for IPA was a culture from sputum or tracheal aspirate from the lower respiratory tract were deemed to represent colonization (18).

Patients were excluded if they had a history of IPA and exhibited pulmonary aspergillosis. Research approval was granted by the institutional review board of Wuhan Union Hospital, as the central coordinating center (approval number 2020-0041-1). The requirement for written for informed consent was waived.

### Data Collection

Patient identification in the ICUs was achieved by reviewing admission logs from available medical records as previously described (2, 7, 19). Data were extracted from local servers by experienced research physicians at each center. Patients' demographic data, preexisting comorbidities, vital signs at ICU admission, laboratory values at the time of CAPA diagnosis, galactomannan antigenemia data, microbiology findings, and data pertaining to secondary bacterial infection, complications, and known IPA risk factors including immunosuppressive drugs (steroids and others) were analyzed. With respect to discharged patients, phone calls were made by 15 January 2021 to determine their living status. Mortality at 180 days after the diagnosis of CAPA was analyzed.

### Definitions

Secondary pulmonary bacterial infection was diagnosed if a patient returned a positive culture of a new pathogen from a lower respiratory tract pathology specimen (bacterial pneumonia) acquired ≥48 h after ICU admission (2). Neutropenia was defined as an absolute neutrophil count of <0.5 × 10^9^/L within the 3 days before the diagnosis of IPA. European Organization for Research and Treatment of Cancer/Invasive Fungal Infections Cooperative Group and the National Institute of Allergy and Infectious Diseases Mycoses Study Group (EORTC/MSG)-host factors were identified as previously described (18). Thrombocytopenia is defined as platelets less than 125 × 10^9^/L (2, 19).

### Statistical Analysis

Due to the exploratory nature of the study as many eligible cases as possible were included. Values were expressed as means ± the standard deviation, medians and interquartile ranges (IQRs), or medians and ranges for continuous variables, and as numbers and percentages for categorical variables. Differences between patients with and without CAPA were investigated using the two-sample *t*-test for parametric variables, the Wilcoxon rank-sum test for non-parametric variables, and Fisher's exact test for categorical variables. To explore independent risk factors for the development of CAPA, age, lymphocyte and platelet counts, treatments, comorbidities, and dichotomous complications associated with a *p*-value of < 0.1 in univariate analyses were included in Cox proportional hazards regression analysis. Age was dichotomized at 60 years, lymphocyte counts at the median value, and platelet counts at 125 × 10^9^/L. Survival probabilities 180 days after the diagnosis of CAPA were analyzed *via* Kaplan-Meier curves, and compared using log-rank tests. Tests were two-sided, and *p* < 0.05 was deemed to indicate statistical significance. Stata/IC 15.1 software (StataCorp, College Station, TX, USA) was used for all analyses.

## Results

### Clinical Characteristics and Test Results

**Figure 1:**
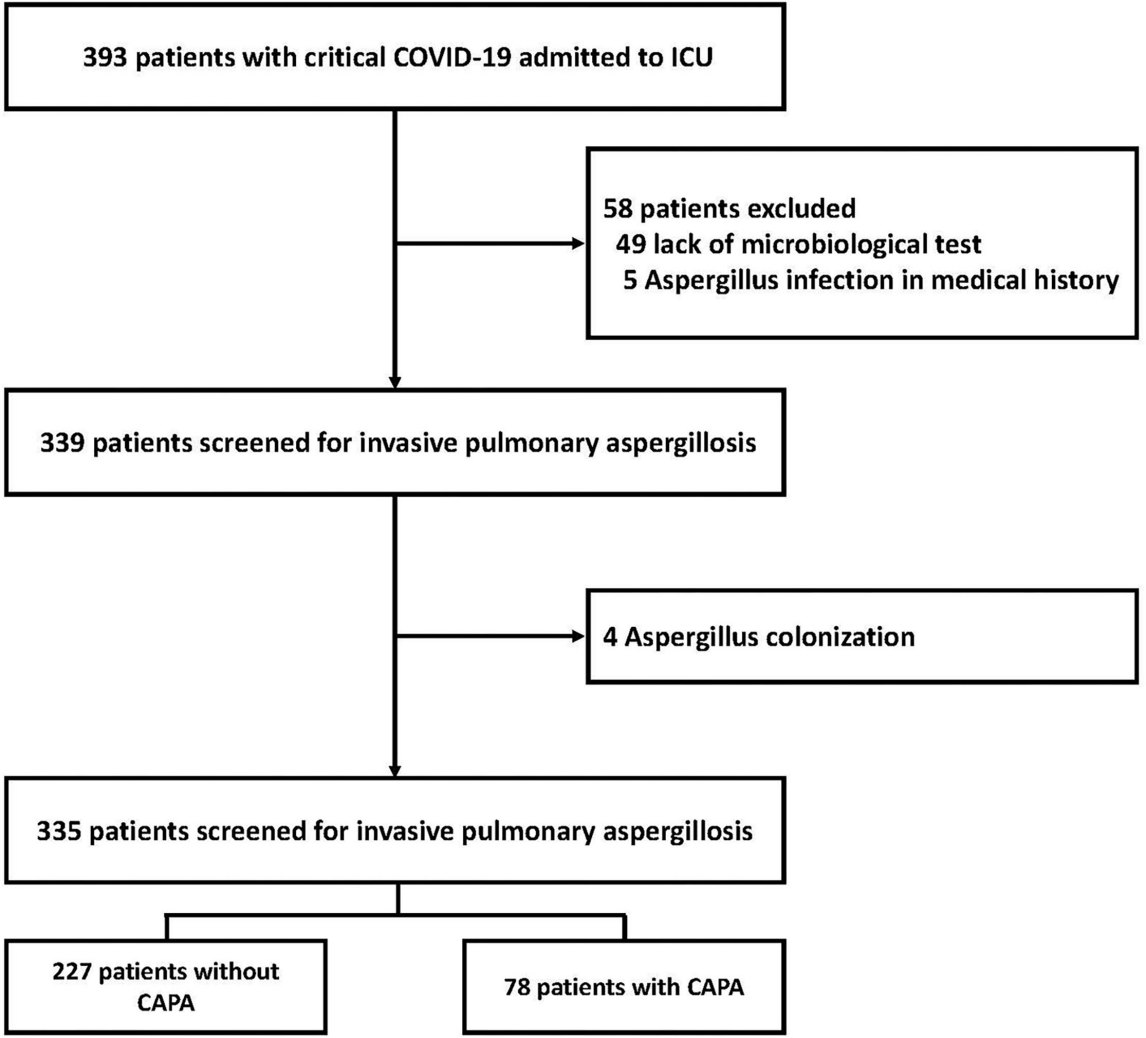
Flowchart of the study including patients with CAPA. CAPA, coronavirus disease 2019-associated invasive pulmonary aspergillosis; COVID-19, coronavirus disease 2019.

A total of 393 COVID-19 patients admitted to ICUs at the six participating centers and screened between 31 December 2019 and 01 April 2020 were initially considered for inclusion in the final analysis. Fifty-eight patients were subsequently excluded; 49 due to unavailability of sufficient information pertaining to microbiological testing while in the ICU, 5 with a medical history of IPA, and 4 with *Aspergillus* spp. colonization. The final analysis set thus included 335 patients with COVID-19 (Figure 1). Their mean age was 60.1 ± 14.1 years, 191 (57%) were male, and 25 (7.5%) were EORTC/MSG host-factor positive. The mean acute physiology and chronic evaluation II score was 15 (IQR 12–19). Based on the CAPA definition applied, 78 (23.3%) cases were diagnosed a median 20.5 days after COVID-19 symptom onset (IQR 13.0–42.0 days). BAL culture tests were performed in 62/78 (79.5%) patients with CAPA, 35 (44.9%) of which were positive for *Aspergillus spp*. Serum galactomannan tests were performed in 66/78 (84.6%) CAPA patients, and were positive as determined via an optical density ≥ 0.5 in 49 patients (62.8%) (Table 1).

**Table 1:** Invasive pulmonary aspergillosis characteristics of COVID-19 patients admitted to ICU.

	**CAPA *n* = 78(%)**
ICU admission to first cultures or GM tests, days^¶^	0 [0-1]
Mycological tests	
Day of first indication after ICU admission	1 [0-3]
Day of first determination after ICU admission	1 [0-9]
Cultures	62 (79.5)
BAL culture positive	36 (46.2)
*Aspergillus fumigatus*	21 (28.8)
*Aspergillus flavus*	15 (20.6)
*Aspergillus niger*	1 (1.4)
Serum GM tests	66 (84.6)
Positive serum GM (index > 0.5)	49 (62.8)
Serum GM value, index, median (IQR)^#^	0.41 [0.12-1.01]
BAL GM	12 (15.4)
Positive BAL GM (index > 1.0)	9 (75.0)
BAL GM value, index, median (IQR)^*^	2.30 [1.25-5.32]
Aspergillus PCR test	15 (19.2)
Positive Aspergillus PCR	3 (20)
Time from illness onset to CAPA diagnosis	20.5 [13.0-42.0]

*BAL, bronchoalveolar lavage; COVID-19, coronavirus disease 2019; CAPA, coronavirus-associated invasive pulmonary aspergillosis; GM, galactomannan; ICU, intensive care unit; PCR, polymerase chain reaction; IQR, interquartile range.*

¶ *Days of first cultures or GM tests after ICU admission, the tests were available in 78 patients.*

# *Serum GM tests were conducted in 66 patients.*

* *BAL GM value was from the 9 patients with GM index > 1.0.*

*Data were expressed as count (%) unless otherwise*.

Differences in characteristics, risk factors, and outcome parameters between patients with and without CAPA are shown in Table 2. A greater proportion of CAPA patients were aged > 60 years (71.8 vs. 52.5%, *p* = 0.002). There were no significant differences in underlying diseases between patients with and without CAPA, with the exception of hypertension (*p* = 0.046). Compared with patients without CAPA, more patients with CAPA had lymphocyte counts < 0.8 × 10^9^/L (76.9 vs. 56.8%, *p* = 0.047) and platelet counts < 125 × 10^9^/L (50 vs. 19.5%, *p* < 0.001). Patients with CAPA were more likely to have secondary bacterial infections (15.4 vs. 6.2%, *p* = 0.013).

**Table 2:** Demographic characteristics, underlying diseases, and clinical characteristics of COVID-19 patients with or without CAPA.

	**Total *N* = 335 (%)**	**With CAPA *N* = 78 (%)**	**Without CAPA** ***N*** **= 257 (%)**	***P*** **-value**
**Demographics**				
Age, years, mean (±SD)	60.1 ± 14.1	64.3 ± 13.6	58.8 ± 14.0	0.0024
Age ≥ 60 years	191 (57.0)	56 (71.8)	135 (52.5)	0.002
Male	198 (59.1)	49 (62.8)	149 (57.9)	0.265
APACHE II admission^*^	15 [12-19]	16 [15-21.5]	15 [12-19]	0.0076
SOFA admission^*^	5 [4-7]	5 [4-8]	5 [4-6]	0.0325
**Underlying diseases**				
Any	197 (58.1)	56 (71.8)	141 (54.9)	0.005
Hypertension	129 (38.5)	38(48.7)	91 (35.4)	0.046
Diabetes mellitus	63 (18.1)	16(20.5)	47 (18.3)	0.385
Coronary disease	28 (8.4)	8 (10.3)	20 (7.8)	0.314
Chronic cardiac disease	37 (11.0)	13 (16.7)	24 (9.3)	0.058
Autoimmune disease	3 (0.9)	2 (3.3)	1 (0.4)	0.137
Cerebrovascular disease	37 (11.0)	10 (12.8)	27 (10.5)	0.542
Chronic liver disease	22 (6.6)	5 (6.4)	17 (6.6)	0.593
Liver cirrhosis	3 (0.9)	1 (1.3)	2 (0.8)	0.550
Chronic kidney disease	18 (5.4)	3 (3.9)	15 (5.8)	0.362
Chronic pulmonary disease	14 (4.2)	5 (6.4)	9 (3.5)	0.206
**Symptoms at hospital admission**				
Fever	276 (82.3)	65 (83.3)	211 (82.1)	0.476
>38°C	21 (6.3)	5 (6.4)	16 (6.2)	0.566
Cough	237 (70.8)	60 (76.9)	177 (68.9)	0.109
Dyspnoea	122 (36.4)	35 (44.9)	87 (33.9)	0.052
**Known risk factors**				
EORTC/MSG host factor	25 (7.5)	9 (11.5)	16 (6.2)	0.097
Malignancy	16 (4.8)	3 (3.9)	13 (5.1)	0.466
Human immunodeficiency virus	4 (0.9)	2 (2.6)	4 (0.8)	0.137
Immunodeficiency	42 (12.5)	9 (11.5)	33 (12.8)	0.467
Neutropenia (<0.5 × 10^9^/L)	1 (0.3)	1 (1.3)	0 (0)	0.233
COPD	14 (4.2)	4 (5.1)	10 (3.9)	0.418
Chronic intermittent hemodialysis	9 (2.7)	2 (2.6)	7 (2.7)	0.649
**Studied risk factors**				
Methylprednisolone 7 days before ICU	46 (13.7)	11 (14.1)	35 (13.6)	0.522
CS 7 days before fungal	63 (18.8)	19 (24.4)	44 (17.1)	0.104
Corticosteroid duration, d	7 [4-13]	7 [4-14]	7 [4-12]	0.4397
>5	74 (66.7)	27 (69.2)	47 (65.2)	0.419
**Laboratory tests at admission**				
Leukocyte count, cells/μl	7.6 [5.1-11.9]	8.2 [5.2-12.7]	7.2 [5.1-11.4]	0.2013
Leukocyte count < 4.0 × 10^9^/L	33 (9.9)	6 (7.7)	27 (10.5)	0.313
Neutrophils, 10^9^/L	6.3 [3.7-10.6]	7.1 [4.2-11.2]	5.9 [3.5-9.9]	0.1150
Neutrophils < 1.5 × 10^9^/L	18 (5.4)	5 (6.4)	13 (5.1)	0.412
Lymphocytes, 10^9^/L**^†^**	0.8 [0.5-1.1]	0.7 [0.5-1.1]	0.8 [0.5-1.1]	0.2279
Lymphocytes < 0.8 × 10^9^/L	206 (61.5)	60 (76.9)	146 (56.8)	0.001
Platelets, 10^9^/L^‡^	186 [144-254]	165 [116-219]	193 [150-261]	0.0013
Platelets < 150 × 10^9^/L	89 (26.6)	39 (50.0)	50 (19.5)	<0.001
Creatinine, mg/dl	71.2 [57.5-87.3]	71.5 [54.4-103.0]	71.2 [58.4-85.7]	0.5072
C-reactive protein, mg/dl	42 [13.8-116.0]	79.3 [19.7-152.8]	34.0 [9.3-97.9]	0.0185
C-reactive protein> 250 μ/L	170 (50.8)	50 (64.1)	120 (46.7)	0.005
LDH, IU/L	354 [263-487]	398 [294-574.5]	325 [251-464]	0.0063
LDH > 250 μ/L	257 (76.7)	64 (82.1)	193 (75.1)	0.130
IL-6, pg/ml	8.9 [6.7-13.4]	10.2 [6.8-16.1]	8.7 [6.6-12.5]	0.0787
Elevated IL-6 level^¶^	251 (74.9)	60 (76.9)	191 (74.3)	0.381
**Secondary bacterial infection at time of diagnosis**				
Sputum or BAL fluid	28 (8.4)	12 (15.4)	16 (6.2)	0.013
Gram-negative bacteria, *n* (%)
Respiratory nonfermenting bacteria^c^	28 (8.4)	12 (15.4)	16 (6.2)	0.013
Pseudomonas spp.	2 (7.4)	1 (1.3)	1 (0.4)	0.412
Gram-positive bacteria, *n* (%)	0 (0)	0 (0)	0 (0)	
Blood	17 (5.1)	6 (7.7)	11 (4.3)	0.179
Hydrothoraxa	4 (1.2)	2 (2.6)	2 (0.8)	0.232
**Fungus culture results^⁋^**				
*Candida* sp., *n* (%)	75 (22.4)	15 (19.2)	60 (23.4)	0.275
*Mucormucedo* sp., *n* (%)	1 (0.3)	1 (1.3)	0 (0.0)	0.233
**Treatment before CAPA diagnosis**				
Commutative dosages, median [IQR], mg	440 [230–680]	480 [240–740]	430 [180–660]	0.4550
Dose, methylprednisolon equivalent/days, mg	61.1 ± 23.3	61.8 ± 24.6	60.7 ± 22.8	0.6595
≥20	130 (38.8)	42 (53.9)	88 (34.2)	0.002
≥40	27 (8.1)	8 (10.3)	19 (7.4)	0.04
Duration of methylprednisolon, days	7 [4-12]	8 [4-14]	7 [4-11]	0.2094
Tocilizumab	6 (1.8)	3 (3.9)	3 (1.2)	0.141
Thymosin α1	159 (47.5)	37 (47.4)	122 (47.5)	0.550
**Organ support at time of diagnosis**				
Vasopressors use	51 (15.2)	29 (37.2)	22 (8.6)	<0.001
Mechanical ventilation	142 (42.4)	46 (59.0)	96 (37.4)	0.001
Non-invasive	82 (24.5)	21(26.9)	61 (23.7)	0.332
Non-invasive mechanical ventilation, days	5 [2-8]	8 [2-15]	5 [2-7]	0.2089
Invasive	137 (40.9)	45 (57.7)	92 (35.8)	<0.001
Invasive mechanical ventilation, days	10 [4-20]	15 [8-26]	7 [3-15]	0.002
Renal replacement therapy, *n* (%)	64 (19.1)	29 (37.2)	35 (13.6)	<0.001
Renal replacement therapy days	5 [2-9.5]	6 [2-9]	3 [1-10]	0.3097
**Outcome**				
Alive at ICU discharge, *n* (%)	221 (66.0)	37 (47.4)	184 (71.6)	<0.001
Length of ICU stay, days	13 [8-20]	17 [10-29]	12 [8-17]	0.007
Length of hospital stay, days	15 [9-24]	21 [15-33]	13 [9-21]	<0.001
ICU mortality	114 (34.0)	41 (52.6)	73 (28.4)	<0.001
180-day mortality	137 (40.9)	51 (65.4)	86 (33.5)	<0.001

*APACHE II, Acute Physiology and Chronic Health Evaluation II; CAPA, coronavirus-associated invasive pulmonary aspergillosis; COPD, chronic obstructive pulmonary disease; COVID-19, coronavirus disease 2019; HFNC, High-flow nasal cannula; ICU, intensive care unit; IQR, interquartile range; IL 6, interleukin 6; LDH, lactate dehydrogenase; pO_2_, partial pressure of oxygen; FiO_2_, fraction of inspired oxygen; pCO_2_, partial pressure of carbon dioxide; SOFA, sequential organ failure assessment.*

*Data were expressed median [interquartile range] or as mean ± standard deviation.*

* *Scores at ICU admission were available in 155 patients, because arterial blood gas analysis was conducted in 103 non-CAPA and 52 CAPA.*

† *The lower limit of normal range of lymphocyte count was 1.1 × 10^9^/L.*

‡ *The lower limit of normal range of platelet count was 125 × 10^9^/L.*

¶ *The upper limit of normal range was 7 pg/ml.*

⁋ *The fungus was from the culture of tracheal aspirate or sputum.*

c *Pseudomonas spp., Acinetobacter spp., Stenotrophomonas spp., Burkholderia spp., Escherichia coli*.

Compared to those without CAPA, patients with CAPA were more likely to require vasopressors (37.2 vs. 8.6%, *p* < 0.001), mechanical ventilation support (57.7 vs. 35.8%, *p* < 0.001), and renal replacement therapy (37.2 vs. 13.6%, *p* < 0.001). There were no significant differences in mean daily methylprednisolone equivalent dose (61.8 ± 24.6 mg vs. 60.7 ± 22.8 mg, *p* > 0.6) or the duration of methylprednisolone (8 days vs. 7 days, *p* > 0.2) between the two groups. Patients with CAPA were significantly more likely to use a higher daily dose (≥40 mg) of methylprednisolone than those without CAPA (53.9 vs. 34.2%, *p* = 0.002) (Table 2).

### Risk Factors for CAPA Development

Data pertaining to risk factors for the occurrence of CAPA derived from pooled data from all patients are shown in Table 3. Thrombocytopenia (HR 1.98, 95% CI 1.16–3.37, *p* = 0.012), vasopressor use (HR 3.57, 95% CI 1.80–7.06, *p* < 0.001), and the use of methylprednisolone at a daily dose of ≥40 mg (HR 1.69, 95% CI 1.02–2.79, *p* = 1.02–2.79) before CAPA diagnosis were independently associated with an increased risk of developing CAPA. Conversely, EORTC/MSG host factor, a lymphocyte count lower than the median of all patients included in the analysis, renal replacement therapy, and secondary pulmonary bacterial infection were not.

**Table 3:** Risk factors for the development of CAPA in critically ill patients with COVID-19.

**Variables**	**HR (95% CI)**	***P*** **-value**
Age ≥ 60	1.16 (0.67-2.01)	0.596
Underlying disease	1.33 (0.78-2.27)	0.302
EORTC/MSG host factor	1.03 (0.49-2.18)	0.486
Lymphocyte count < 0.8 × 10^9^/L**^†^**	1.34 (0.74-2.45)	0.337
Thrombocytopenia^‡^	1.98 (1.16-3.37)	0.012
C-reactive protein	1.40 (0.87-2.27)	0.166
Vasopressors use	3.57 (1.80-7.06)	<0.001
Corticosteroid dosage		
≥20 mg/d	0.34 (0.04-2.56)	0.292
≥40 mg/d	1.69 (1.02-2.79)	0.043
Renal replacement therapy	0.79 (0.37-1.71)	0.552
IMV	0.65 (0.34-1.24)	0.189
Secondary bacterial infection	1.35 (0.68-2.69)	0.392

*CAPA, coronavirus-associated invasive pulmonary aspergillosis; COVID-19, coronavirus disease 2019; CI, confidence interval; HR, hazard ratio; IMV, invasive mechanical ventilation.*

† *The low limit of normal range of lymphocyte count was 1.1 × 10^9^/L.*

‡ *The low limit of normal range of platelet count was 125 × 10^9^/L*.

### Clinical Outcomes

**Figure 2:**
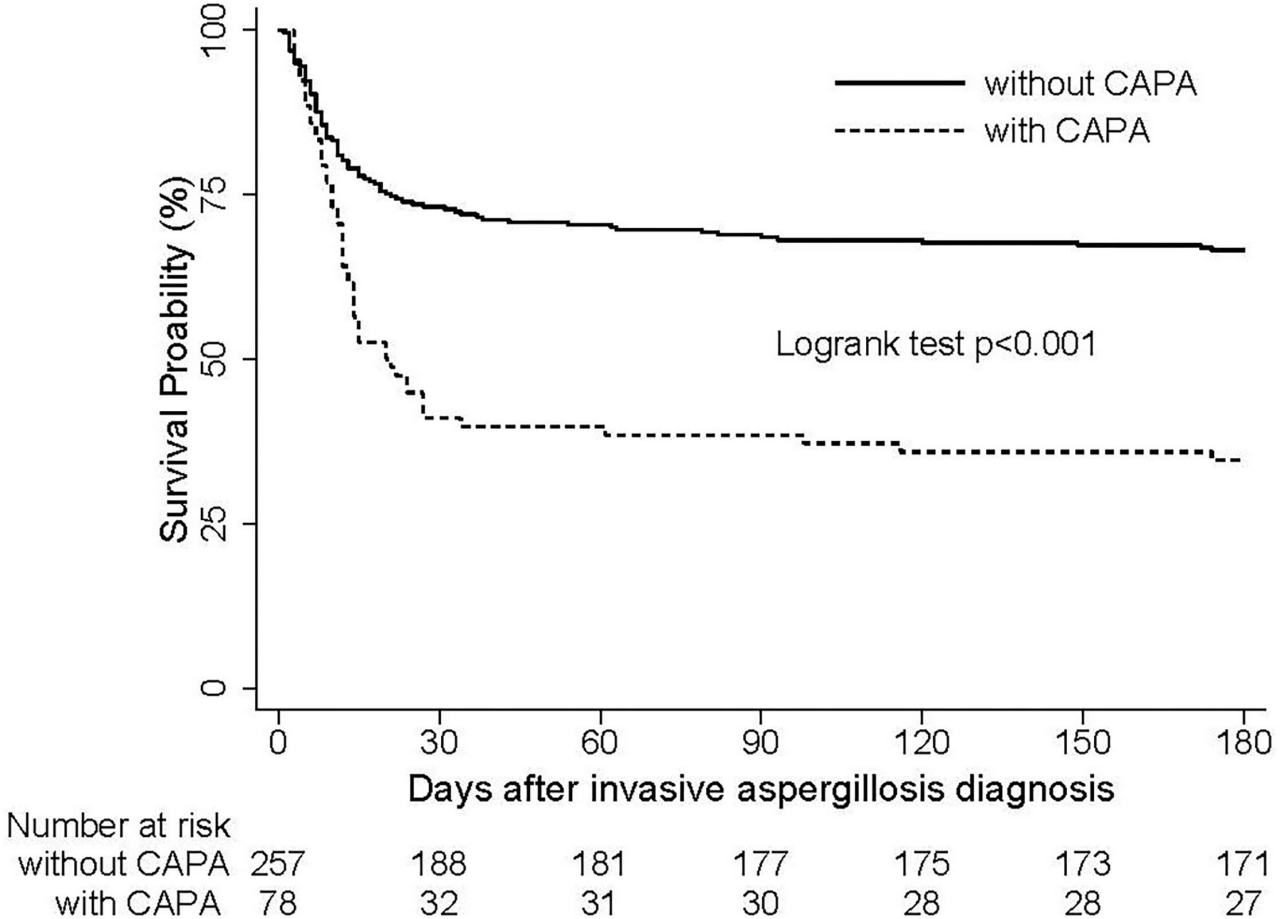
Survival curves of 335 critically ill patients with or without CAPA. The number of patients with CAPA was 78, and the number without CAPA was 257. CAPA, coronavirus disease 2019-associated invasive pulmonary aspergillosis; COVID-19, coronavirus disease 2019.

All patients returned negative CAPA test results before they were discharged. Median ICU stays were significantly longer in CAPA patients than in those without CAPA (17 days vs. 12 days, *p* = 0.007), as were median hospital stays (21 days vs. 13 days, *p* < 0.001). ICU mortality was higher in CAPA patients than in those without CAPA (52.6 vs. 28.4%, *p* < 0.001). At 180 days after the initial diagnosis of CAPA no patients tested positive for CAPA and were still being hospitalized, and 51 (65.4%) patients with CAPA had died. Kaplan-Meier curves depicted significantly higher 180-day mortality after aspergillosis diagnosis in CAPA patients compared with patients without CAPA (*p* < 0.001) (Figure 2).

## Discussion

Early identification of CAPA in critically ill COVID-19 patients admitted to ICUs will facilitate the optimal use of medical resources. The current multicenter retrospective study investigated risk factors associated with CAPA, and long-term outcomes in COVID-19 patients admitted to ICUs. Patients with thrombocytopenia, vasopressor use before CAPA diagnosis, and the use of methylprednisolone at a daily dose of ≥40 mg were much more likely to develop CAPA.

The reported incidences of CAPA in patients admitted to ICUs vary widely from 2.5 to 39.1%, and in the current cohort it was 23.3% (8, 9, 20, 21). Differences may be associated with different diagnostic approaches or interpretations of IPA markers (3). BAL culture and galactomannan testing of BAL are a gold standard for IPA diagnosis, but BAL and autopsy are usually lacking in patients with COVID-19 due to fear of viral particles spreading. In the present cohort BAL culture was only performed in 62 (79.5%) CAPA patients, and in 48.7% of patients the BAL cultures were *Aspergillus* spp.-positive. Given the limited sensitivity of BAL *Aspergillus* spp. culture (13, 22–24), a serum galactomannan test—which was performed in 316 (94.3%) of 335 patients including 66 (84.6%) of 78 patients with CAPA—was used to classify patients in the present study. Of the patients with CAPA in the current cohort 61.5% yielded positive serum galactomannan tests.

In a study of 630 patients with influenza in the ICU, invasive aspergillosis was detected in 17%. The slightly lower incidence in an influenza cohort could be explained by the different pathogenesis of SARS-CoV-2 infection (18). In most influenza patients respiratory epithelium damage and mucociliary clearance dysfunction may facilitate invasion by *Aspergillus* spp. Unlike influenza however, in addition to respiratory system damage SARS-CoV-2 sends parts of the immune system into overdrive or depletes certain immune cells, impairing the lung's capacity to clear *Aspergillus* spp. or leaving the patient less able to fight off other infections (3, 6).

Steroids such as dexamethasone (6 mg daily dose) have been shown to curb overactive immune responses and improve survival rates in severely ill COVID-19 patients (25), but steroids are a double-edged sword because they can render the patient susceptible to other infections, particularly *Aspergillus* spp. In the current cohort the use of ≥40 mg of methylprednisolone (equivalent to 7.5 mg of dexamethasone) before CAPA diagnosis was independently associated with the development of invasive pulmonary aspergillosis, consistent with corticosteroid treatment increasing the risks of hyperglycemia and infection in severe COVID-19 (15). In a recent observational study COVID-19 patients received higher than recommended steroid doses and were likely have *Aspergillus* spp. infections (26), therefore IPA infections in critically ill COVID-19 patients should be inversely related to the dosage of immunosuppressive therapy.

Platelets have versatile antifungal immune functions, and thrombocytopenic hosts are highly susceptible to IPA and can rapidly succumb to infection (27–29). To the best of our knowledge no study has investigated the correlation between thrombocytopenia in COVID-19 patients and IPA susceptibility. In the present study thrombocytopenia was an independent risk factor for CAPA, but the underlying mechanisms are not clear. Mechanisms of IPA-related thrombocytopenia have been suggested (27–29). One is that when infected with *Aspergillus* spp. the galactosaminogalactan secreted by the pathogen is deposited on the surfaces of platelets, inducing their activation and resulting in fungal damage (27, 29, 30). Another is that the altered platelet surface triggers a complement cascade reaction that attracts and activates phagocytes to eliminate the invading *Aspergillus* spp. (27, 28). In the current cohort platelets were extremely low in critically ill patients with COVID-19, thus their susceptibility to IPA was increased.

In the present study mortality in patients with CAPA was 65.4% at 180 days after CAPA diagnosis, and the study is the first report on the long-term outcomes of CAPA. The rate is higher than that previously reported in patients admitted to the ICU with influenza-associated IPA at 90 days (51%) (18), and in other cohorts such as aspergillosis patients with phlebovirus (~40%) (31). The different incidence may be because the pathophysiology of COVID-19 differs substantially from that of influenza and phlebovirus infections. On 04 April 2021 the European Centre for Disease Prevention and Control (CDC) stated that in 186 patients with CAPA the mortality rate was 52.2%, which was similar to the ICU mortality in the current study (52.6%). The study was somewhat cross-sectional in that it included data at 6 and 12 weeks after CAPA diagnosis. The number of patients with CAPA had decreased by the 90-day timepoint because a lot of them had died by that time. In the present cohort, a number of patients with CAPA decreased after 90 days. Mortality was also considerably high in case series from France, Germany, Belgium, and the Netherlands, ranging from 44.5–66.7% (10, 11, 13, 32). Of particular importance was the 100% fatality rate of patients with underlying diseases reported in the Netherlands (10).

The current study had several limitations. Due to its retrospective design confounding cannot be ruled out and a standardized diagnostic approach toward IPA was not used. Considerations of mycological criteria and antigen testing were included in case classifications however, and the galactomannan index was beneficial for the early identification of CAPA patients at risk of a poor outcome. Thus, we believe that the diagnosis of CAPA in the study was more accurate and clinically pertinent. Another potential limitation was that 13 patients with only positive serum galactomannan data were categorized as CAPA patients, and this was likely to have included some false-positive cases. A higher serum galactomannan index represents an increased *Aspergillus* spp. burden however, because severe SARS-CoV-2 infection is likely to contribute to *Aspergillus* spp. micro-angioinvasion at the interface of the alveoli and blood vessels. Indeed, there was no difference in mortality between the galactomannan-positive and the galactomannan-negative groups among the CAPA patients in the study. Whether serum galactomannan should be considered as a standardized diagnostic approach should be a focus of future prospective studies.

## Conclusion

Thrombocytopenia, vasopressor use before CAPA diagnosis, and the use of methylprednisolone at a daily dose ≥ 40 mg are independent predictors of the development of CAPA. The occurrence of CAPA increases the long-term mortality of critically ill COVID-19 patients. The results of the current study may help physicians to optimize the management of critically ill COVID-19 patients, and improve antifungal management.

## Data Availability Statement

All datasets generated for this study are included in the article/Supplementary Material. Deidentified participant data will be provided after approval from the corresponding author and study center.

## Ethics Statement

Research approval (2020-0041-1) was granted by the institutional review board of Wuhan Union Hospital as the central coordinating center. Written informed consent from the participants' legal guardian/next of kin was not required to participate in this study in accordance with the national legislation and the institutional requirements.

## Author Contributions

JX, ZL, XZ, LZ, WC, and BL collected the epidemiological and clinical data. JX, XY, and TZ summarized all data. JX, XY, HL, YY, and YS contributed to literature search and writing of the manuscript. YS, CH, SY, and MH designed the study, had full access to all data in the study, and take full responsibility for accuracy of the analyses and their interpretation. All authors contributed to the article and approved the submitted version.

## Funding

The research was designed, conducted, analyzed, and interpreted by the authors entirely independently of the funding sources.

## Conflict of Interest

The authors declare that the research was conducted in the absence of any commercial or financial relationships that could be construed as a potential conflict of interest.

## Publisher's Note

All claims expressed in this article are solely those of the authors and do not necessarily represent those of their affiliated organizations, or those of the publisher, the editors and the reviewers. Any product that may be evaluated in this article, or claim that may be made by its manufacturer, is not guaranteed or endorsed by the publisher.

## References

[1] Karki R Man SM Malireddi RKS Kesavardhana S Zhu Q Burton AR. NLRC3 is an inhibitory sensor of PI3K-mTOR pathways in cancer. Nature. (2016) 540:583-7. 10.1038/nature20597 27951586 PMC5468516

[2] Xu J Yang X Yang L Zou X Wang Y Wu Y. Clinical course and predictors of 60-day mortality in 239 critically ill patients with COVID-19: a multicenter retrospective study from Wuhan, China. Crit Care. (2020) 24:394. 10.1186/s13054-020-03098-9 32631393 PMC7336107

[3] Lamoth F Lewis RE Walsh TJ Kontoyiannis DP. Navigating the uncertainties of COVID-19 associated aspergillosis (CAPA): a comparison with influenza associated aspergillosis (IAPA). J Infect Dis. (2021) 26:jiab163. 10.1093/infdis/jiab163 33770176 PMC8083649

[4] Fekkar A Lampros A Mayaux J Poignon C Demeret S Constantin JM. Occurrence of invasive pulmonary fungal infections in patients with severe COVID-19 admitted to the ICU. Am J Respir Crit Care Med. (2021) 203:307-17. 10.1164/rccm.202009-3400OC 33480831 PMC7874326

[5] White PL Dhillon R Cordey A Hughes H Faggian F Soni S. A national strategy to diagnose COVID-19 associated invasive fungal disease in the ICU. Clin Infect Dis. (2020) 73:e1634–44. 10.2139/ssrn.3644400 PMC7499527 32860682

[6] Koehler P Bassetti M Chakrabarti A Chen SCA Colombo AL Hoenigl M. Defining and managing COVID-19-associated pulmonary aspergillosis: the 2020 ECMM/ISHAM consensus criteria for research and clinical guidance. Lancet Infect Dis. (2021) 21:e149-62. 10.1016/S1473-3099(20)30847-1 33333012 PMC7833078

[7] Yang X Yu Y Xu J Shu H Xia J Liu H. Clinical course and outcomes of critically ill patients with SARS-CoV-2 pneumonia in Wuhan, China: a single-centered, retrospective, observational study. Lancet Respir Med. (2020) 8:475-81. 10.1016/S2213-2600(20)30079-5 32105632 PMC7102538

[8] Lamoth F Glampedakis E Boillat-Blanco N Oddo M Pagani JL. Incidence of invasive pulmonary aspergillosis among critically ill COVID-19 patients. Clin Microbiol Infect. (2020) 26:1706-8. 10.1016/j.cmi.2020.07.010 32659385 PMC7348600

[9] Nasir N Farooqi J Mahmood SF Jabeen K. COVID-19-associated pulmonary aspergillosis (CAPA) in patients admitted with severe COVID-19 pneumonia: An observational study from Pakistan. Mycoses. (2020) 63:766-70. 10.1111/myc.13135 32585069 PMC7361517

[10] van Arkel ALE Rijpstra TA Belderbos HNA van Wijngaarden P Verweij PE Bentvelsen RG. COVID-19-associated Pulmonary Aspergillosis. Am J Respir Crit Care Med. (2020) 202:132-5. 10.1164/rccm.202004-1038LE 32396381 PMC7328331

[11] Alanio A Dellière S Fodil S Bretagne S Mégarbane B. Prevalence of putative invasive pulmonary aspergillosis in critically ill patients with COVID-19. Lancet Respir Med. (2020) 8:e48-9. 10.1016/S2213-2600(20)30237-X 32445626 PMC7239617

[12] Bartoletti M Pascale R Cricca M Rinaldi M Maccaro A Bussini L. Epidemiology of invasive pulmonary aspergillosis among COVID-19 intubated patients: a prospective study. Clin Infect Dis. (2020) 28:ciaa1065. 10.1093/cid/ciaa1065 32719848 PMC7454393

[13] Rutsaert L Steinfort N Van Hunsel T Bomans P Naesens R Mertes H. COVID-19-associated invasive pulmonary aspergillosis. Ann Intensive Care. (2020) 10:71. 10.1186/s13613-020-00686-4 32488446 PMC7265874

[14] Onder G Rezza G Brusaferro S. Case-fatality rate and characteristics of patients dying in relation to COVID-19 in Italy. JAMA. (2020) 323:1775-6. 10.1001/jama.2020.4683 32203977

[15] Cai J Li H Zhang C Chen Z Liu H Lei F. The Neutrophil-to-lymphocyte ratio determines clinical efficacy of corticosteroid therapy in patients with COVID-19. Cell Metab. (2021) 33:258-69.e253. 10.1016/j.cmet.2021.01.002 33421384 PMC7832609

[16] Alhazzani W Møller MH Arabi YM Loeb M Gong MN Fan E. Surviving sepsis campaign: guidelines on the management of critically ill adults with Coronavirus Disease (2019) (COVID-19). Intensive Care Med. (2020) 46:854-87. 10.1007/s00134-020-06022-5 32222812 PMC7101866

[17] Shang Y Pan C Yang X Zhong M Shang X Wu Z. Management of critically ill patients with COVID-19 in ICU: statement from front-line intensive care experts in Wuhan, China. Ann Intensive Care. (2020) 10:73. 10.1186/s13613-020-00689-1 32506258 PMC7275657

[18] Schauwvlieghe A Rijnders BJA Philips N Verwijs R Vanderbeke L Van Tienen C. Invasive aspergillosis in patients admitted to the intensive care unit with severe influenza: a retrospective cohort study. Lancet Respir Med. (2018) 6:782-92. 10.1016/S2213-2600(18)30274-1 30076119

[19] Xu J Yang X Huang C Zou X Zhou T Pan S. A novel risk-stratification models of the high-flow nasal cannula therapy in COVID-19 patients with hypoxemic respiratory failure. Front Med. (2020) 7:607821. 10.3389/fmed.2020.607821 33425951 PMC7793962

[20] Permpalung N Chiang TP Massie AB Zhang SX Avery RK Nematollahi S. COVID-19 associated pulmonary aspergillosis in mechanically ventilated patients. Clin Infect Dis. (2021). 10.1093/cid/ciab223 33693551 PMC7989534

[21] Razazi K Arrestier R Haudebourg AF Benelli B Carteaux G Decousser JW. Risks of ventilator-associated pneumonia and invasive pulmonary aspergillosis in patients with viral acute respiratory distress syndrome related or not to Coronavirus 19 disease. Crit Care. (2020) 24:699. 10.1186/s13054-020-03417-0 33339526 PMC7747772

[22] Arastehfar A Wickes BL Ilkit M Pincus DH Daneshnia F Pan W. Identification of mycoses in developing countries. J Fungi. (2019) 5:90. 10.3390/jof5040090 31569472 PMC6958481

[23] Chindamporn A Chakrabarti A Li R Sun PL Tan BH Chua M. Survey of laboratory practices for diagnosis of fungal infection in seven Asian countries: an Asia Fungal Working Group (AFWG) initiative. Med Mycol. (2018) 56:416-25. 10.1093/mmy/myx066 29036605

[24] Eigl S Hoenigl M Spiess B Heldt S Prattes J Neumeister P. Galactomannan testing and Aspergillus PCR in same-day bronchoalveolar lavage and blood samples for diagnosis of invasive aspergillosis. Med Mycol. (2017) 55:528-34. 10.1093/mmy/myw102 27744310

[25] Horby P Lim WS Emberson JR Mafham M Bell JL Linsell L. Dexamethasone in hospitalized patients with Covid-19. N Engl J Med. (2021) 384:693-704. 10.1056/NEJMoa2021436 32678530 PMC7383595

[26] Mitaka H Perlman DC Javaid W Salomon N. Putative invasive pulmonary aspergillosis in critically ill patients with COVID-19: an observational study from New York City. Mycoses. (2020) 63:1368-72. 10.1111/myc.13185 32965042 PMC7646269

[27] Speth C Rambach G Lass-Flörl C. Platelet immunology in fungal infections. Thromb Haemost. (2014) 112:632-39. 10.1160/TH14-01-0074 24990293

[28] Deshmukh H Speth C Sheppard DC Neurauter M Würzner R Lass-Flörl C. Aspergillus-derived galactosaminogalactan triggers complement activation on human platelets. Front Immunol. (2020) 11:550827. 10.3389/fimmu.2020.550827 33123129 PMC7573070

[29] Tischler BY Tosini NL Cramer RA Hohl TM. Platelets are critical for survival and tissue integrity during murine pulmonary *Aspergillus fumigatus* infection. PLoS Pathog. (2020) 16:e1008544. 10.1371/journal.ppat.1008544 32407390 PMC7252636

[30] Perkhofer S Kehrel BE Dierich MP Donnelly JP Nussbaumer W Hofmann J. Human platelets attenuate Aspergillus species *via* granule-dependent mechanisms. J Infect Dis. (2008) 198:1243-6. 10.1086/591458 18752432 PMC2980866

[31] Bae S Hwang HJ Kim MY Kim MJ Chong YP Lee SO. Invasive pulmonary aspergillosis in patients with severe fever with thrombocytopenia syndrome. Clin Infect Dis. (2020) 70:1491-4. 10.1093/cid/ciz673 31342053

[32] Koehler P Cornely OA Böttiger BW Dusse F Eichenauer DA Fuchs F. COVID-19 associated pulmonary aspergillosis. Mycoses. (2020) 63:528-34. 10.1111/myc.13096 32339350 PMC7267243

