# Supplementary material for: Risk Factors for Invasive Aspergillosis in Patients Admitted to the Intensive Care Unit With Coronavirus Disease 2019: A Multicenter Retrospective Study
Source: Front Med (Lausanne). 2021 Nov 16;8:753659. doi: 10.3389/fmed.2021.753659 (PMC8635191; doi:10.3389/fmed.2021.753659)
Supplement: Supplementary file 1 [file Data_Sheet_1.docx]

**Supplementary information**

**Risk Factors for Invasive Aspergillosis in Patients Admitted to the ICU with COVID-19: A Multicenter Retrospective Study**

Jiqian Xu^1,2,6^, Xiaobo Yang^1^, Zheng Lv^1^, Ting Zhou^1^, Hong Liu^1,2^, Xiaojing Zou^1^, Fengsheng Cao^4^, Lu Zhang^4^, Boyi Liu^5^, Wei Chen^5^, Yuan Yu^1^, Huaqing Shu^1^, Shiying Yuan^1^, Ming Hu^3†^, Chaolin Huang^2†^, You Shang^1,2,6†^

**Figure S1.**

**
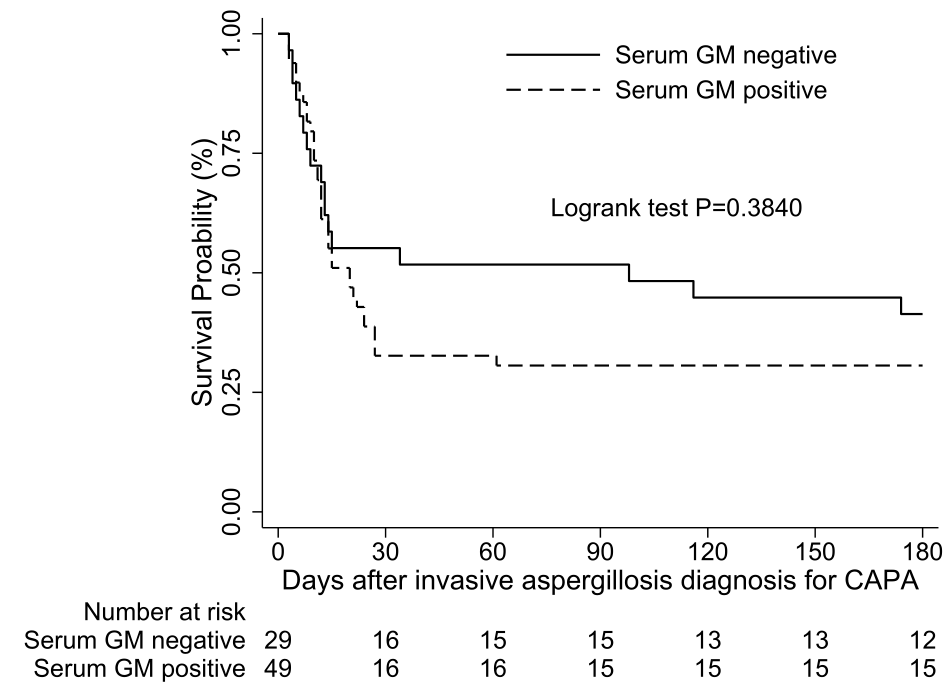
**

**Figure S1. Kaplan–Meier plots of the patients with CAPA according to serum GM index.**

Abbreviations: CAPA: coronavirus disease–associated invasive pulmonary aspergillosis; GM, galactomannan.

**Figure S2.**


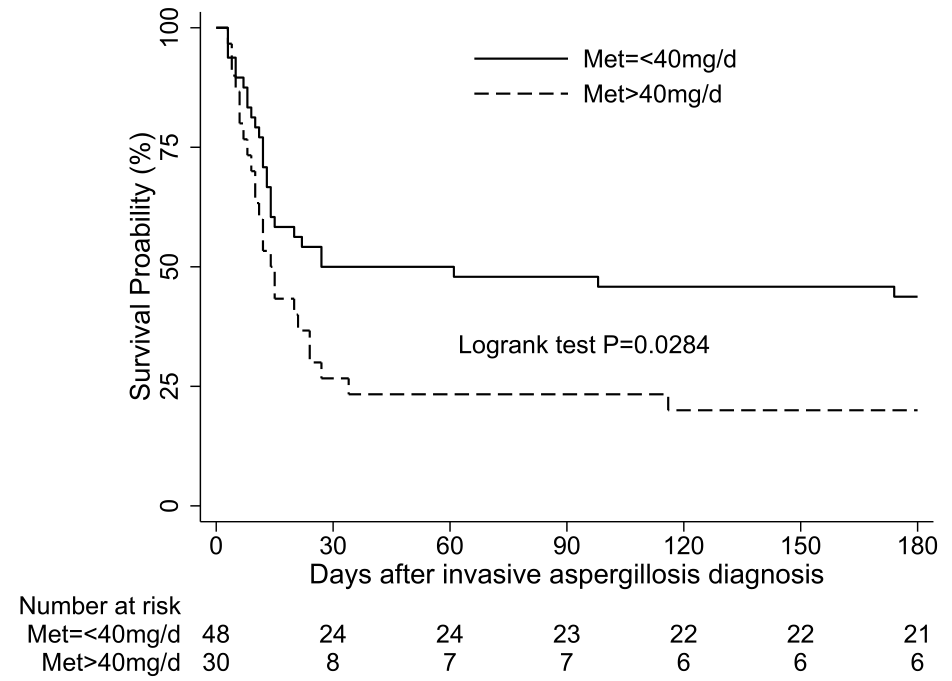


**Figure S2. Survival curve of the patients with CAPA according to Methylprednisolone dosage.**

Abbreviations: CAPA: coronavirus disease–associated invasive pulmonary aspergillosis; Met, Methylprednisolone.

**Table S1. Bacterial Co-infection or Secondary Bacterial Infection of COVID-19 Patients with or without CAPA at the time of CAPA diagnosis***

| **Tracheal aspirate or sputum culture** | **Total**  **N= 28 (%)** | **With CAPA**  **N=12(%)** | **Without CAPA**  **N=16 (%)** | **p value** |
| --- | --- | --- | --- | --- |
| Klebsiella spp. | 13 (46.4) | 10 (62.5) | 3 (25.0) | 0.055 |
| Acinetobacter baumannii | 10 (35.7) | 6 (50.0) | 4 (25.0) | 0.167 |
| Stenotrophomonas maltophilia | 2 (7.1) | 2 (16.7) | 0 (0.0) | 0.175 |
| Pseudomonas spp. | 2 (7.1) | 1 (8.3) | 0 (6.3) | 0.683 |
| Escherichia coli | 1 (3.6) | 0 (0.0) | 1 (6.3) | 0.571 |

**Abbreviations:** CAPA, coronavirus-associated invasive pulmonary aspergillosis; COVID-19, coronavirus disease 2019;

* These bacteria were from the culture of tracheal aspirate or sputum.
